# Alternative Structures of α-Synuclein

**DOI:** 10.3390/molecules25030600

**Published:** 2020-01-30

**Authors:** Dawid Dułak, Małgorzata Gadzała, Mateusz Banach, Leszek Konieczny, Irena Roterman

**Affiliations:** 1 Faculty of Physics, Astronomy and Applied Computer Science, Jagiellonian University, Łojasiewicza 11, 30-348 Kraków, Poland; dawid.dulak@gmail.com; 2 ACK–Cyfronet AGH, Nawojki 11, 30-950 Krakow, Poland; m.k.gadzala@gmail.com; 3 Department of Bioinformatics and Telemedicine, Jagiellonian University—Medical College, Łazarza 16, 31-530 Krakow, Poland; mateusz.banach@uj.edu.pl; 4 Chair of Medical Biochemistry, Jagiellonian University–Medical College, Kopernika 7, 31-034 Kraków, Poland; mbkoniec@cyf-kr.edu.pl

**Keywords:** misfolding, A-synuclein, amyloid, fibril, protein folding, hydrophobicity

## Abstract

The object of our analysis is the structure of alpha-synuclein (ASyn), which, under in vivo conditions, associates with presynaptic vesicles. Misfolding of ASyn is known to be implicated in Parkinson’s disease. The availability of structural information for both the micelle-bound and amyloid form of ASyn enables us to speculate on the specific mechanism of amyloid transformation. This analysis is all the more interesting given the fact that—Unlike in Aβ(1–42) amyloids—only the central fragment (30–100) of ASyn has a fibrillar structure, whereas, its N- and C-terminal fragments (1–30 and 100–140, respectively) are described as random coils. Our work addresses the following question: Can the ASyn chain—as well as the aforementioned individual fragments—adopt globular conformations? In order to provide an answer, we subjected the corresponding sequences to simulations carried out using Robetta and I-Tasser, both of which are regarded as accurate protein structure predictors. In addition, we also applied the fuzzy oil drop (FOD) model, which, in addition to optimizing the protein’s internal free energy, acknowledges the presence of an external force field contributed by the aqueous solvent. This field directs hydrophobic residues to congregate near the center of the protein body while exposing hydrophilic residues on its surface. Comparative analysis of the obtained models suggests that fragments which do not participate in forming the amyloid fibril (i.e., 1–30 and 100–140) can indeed attain globular conformations. We also explain the influence of mutations observed in vivo upon the susceptibility of ASyn to undergo amyloid transformation. In particular, the 30–100 fragment (which adopts a fibrillar structure in PDB) is not predicted to produce a centralized hydrophobic core by any of the applied toolkits (Robetta, I-Tasser, and FOD). This means that in order to minimize the entropically disadvantageous contact between hydrophobic residues and the polar solvent, ASyn adopts the form of a ribbonlike micelle (rather than a spherical one). In other words, the ribbonlike micelle represents a synergy between the conformational preferences of the protein chain and the influence of its environment.

## 1. Introduction

A-synuclein (referred to as ASyn in our work) is strongly expressed in brain tissue [1], particularly at the presynaptic termini [2], and in synaptic membranes [3]. It is also found on the tips of nerve cells (neurons) in specialized structures called presynaptic terminals [4]. The ASyn chain is sometimes divided into an N-terminal fragment (1–60), a NAC (non-amyloid beta component; 61–95) and a strongly hydrophilic C-terminal fragment (96–140) [5]. Another characteristic property of ASyn is the presence of imperfect KTKEGV repeats starting at positions 10, 21, 32, 43, 58, 69, and 80, respectively. The first 25 residues of the N-terminal fragment are responsible for anchoring the protein in the lipid bilayer, whereas, residues 26–98 mediate affinity towards the membrane, depending on the composition of the membrane itself [6]. This affinity is likely related to the biological activity of ASyn, which, however, remains poorly understood (see References [7,8] for further information). The amyloid form of ASyn (PDB ID: 2N0A [9]) comprises a fibrillar central fragment (30–100), while the remaining N- and C-terminal fragments both adopt random coil conformations. The presented work provides an analysis of the entire polypeptide, as well as of each of its fragments. The presence of relatively long fragments which do not contribute to the fibrillar structure distinguishes ASyn from among other amyloids (particularly Aβ [10,11,12,13], and tau [14] amyloids where the amyloid-like structure encompasses the entire chain [9,15]).

Micelle-bound (1XQ8) [16] and fibrillar (2N0A) forms of ASyn were subjected to analysis based on the fuzzy oil drop model (FOD) [17,18,19]. The model assumes that the distribution of hydrophobicity in a globular molecule can be mathematically modeled as a 3D Gaussian, with hydrophobicity peaking at the center of the protein body and decreasing along with the distance from the center, reaching almost 0 on the surface and beyond it. Unlike globular proteins, amyloids follow an entirely different structural pattern, presenting alternating bands of high and low hydrophobicity along their main axis. The fuzzy oil drop model provides a reference, which enables us to determine whether—And to what extent—Any given protein follows the spherical [15] or ribbon-like (linear) pattern [15]. In addition, in the presented work, we also try to determine whether the ASyn chain may adopt a globular form, and thus, become soluble. In order to answer this question, we apply specialized software toolkits—Robetta [17] and I-Tasser [18], both noted for their accuracy in recent editions of the CASP challenge [19]. Additionally, we perform calculations based on the fuzzy oil drop model, enabling us to acknowledge the effects of an external force field (contributed by the aqueous solvent) in addition to internal force fields (atom-atom interactions). This external field directs hydrophobic residues to congregate at the center of the molecule, while promoting exposure of hydrophilic residues [20,21,22]. Consequently, it favors the formation of a globular protein. Taken together, the three software frameworks enable us to generate a diverse spectrum of models [23,24,25,26].

## 2. Results

### 2.1. Parameters Used for Structure Description

The detailed description of the model is given in Materials and Methods. The basic assumptions are given here to make the interpretation of the results easier.

The molecule under consideration is encapsulated in ellipsoid (3D Gauss function). The values of Gauss function in particular points are treated as idealized hydrophobicity level. It is the consequence of the assumption that the protein molecule follows more or less construction of the spherical micelle exposing polar groups on the surface and hiding the hydrophobic residues in the central part of protein body (hydrophobic core). The hydrophobicity distribution is treated as idealized one-called T in this paper. On the other hand, the observed hydrophobicity distribution-the result of hydrophobic interaction depends on the intrinsic hydrophobicity of interacting residues and on the distance between them. This interaction calculated according to Levitt’s function [27] is called as O-observed one. Comparison of these two distributions allows the identification of similarities/differences between them, revealing the status of protein under consideration. The quantitative measurements of differences are possible using the Kullback-Leibler divergence entropy [28] called *D_KL_*. Distance between O and T expressed by *D_KL_* cannot be interpreted (entropy category). This is why the second reference distribution is introduced: The unified one, where each residue is attributed by equal *hydrophobicity level* = 1/N, where N is the number of residues. This distribution called R represents the status of protein molecule deprived of any form of hydrophobicity level differentiation-opposite to T distribution representing the presence of centric hydrophobic core. This is why two *D_KL_* values describe the status of each residue: For the relation of O distribution versus two reference distributions: T and R. The relation between values *D_KL_* for status: O|T and O|R characterizes the O distribution. If *D_KL_* for O|*T* > *O*|R it means that the O distribution is closer to R distribution. In the opposite situation, the presence of a hydrophobic core is assumed to be present in the protein under consideration. To avoid dealing with two parameters, the *RD* (Relative Distance) is introduced expressed as *D_KL_* for O|T divided by the sum of *D_KL_* for O|T and O|R. The RD value lower than 0.5 suggests the presence of a hydrophobic core. This is why this parameter is used to characterize the status of proteins and models (as calculated using special programs: I-TASSER [29], ROBETTA [30]) and FOD model [26] folding protein in the presence of external force field in the form of 3D Gauss function to express the influence of water environment. The models representing the status of *RD* < 0.5 are treated as globular with a hydrophobic core. The detailed description of the procedures is given in Materials and Methods (chapter 4).

The aim of the calculations presented in this paper is to check the specificity of amino acids sequence present in ASyn and particular its sequence in three fragments of the chain (1–30, 30–100 and 100–140) representing different structural forms (only fragment 30–100 in amyloid form). The commonly known assumption is that the amino acids sequence determines the 3D structure of the protein. The question is what specificity of sequence promotes the generation of the spherical micelle and what is the specificity of sequence promoting ribbon-like micelle, which is observed in amyloids [31,32].

Programs predicting 3D structure using different force field are assumed to produce different 3D structures of ASyn and its chain fragments. The analysis of these models is assumed to reveal the specific influence of amino acid sequences on 3D structure promoting the amyloid forms.

### 2.2. Structure of Human Micelle-Bound Alpha-Synuclein (1XQ8)

The structure of ASyn in its micelle-bound form (1XQ8) was subjected to FOD analysis in two approaches, the first of which covered the entire micelle-bound chain, while the second was limited to the dual helix system (1–95) and excluded the loose C-terminal random coil (96–140).

**Figure 1:**
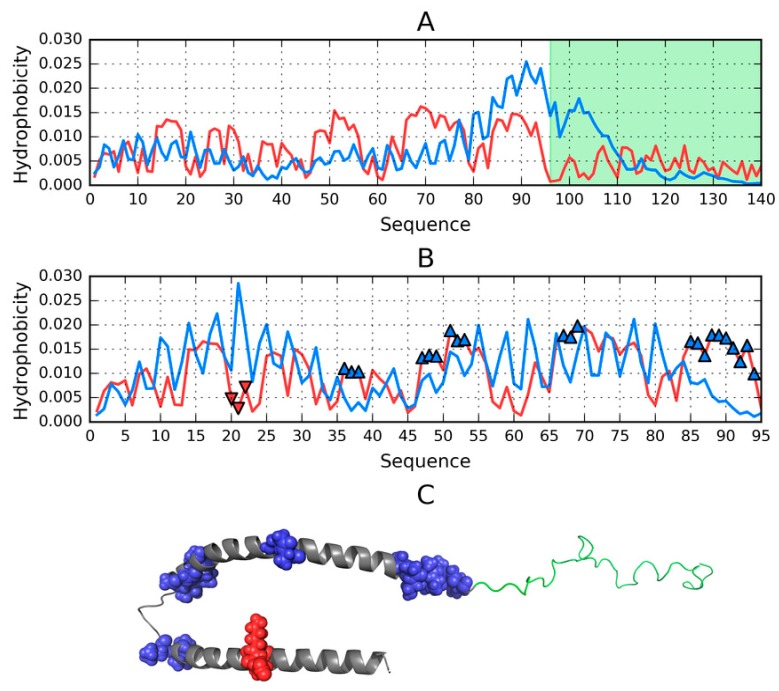
Structure of ASyn in its micelle-bound form (1XQ8); (**A**)—Theoretical (T, blue) and observed (O, red) hydrophobicity density distribution profiles calculated for the complete chain (1–140). Random coil C-terminal fragment (96–140) is highlighted in green, (**B**)—Theoretical (T, blue) and observed (O, red) hydrophobicity density distribution profiles calculated for the 1–95 fragment treated as an individual unit. Blue markers indicate residues No. 36–38, 47–49, 51–53, 67–69, 85–94 which exhibit local excess of hydrophobicity, while red markers correspond to local hydrophobicity deficiency (residues No. 20–22); (**C**)—3D presentation with color-coding corresponding to figures A and B.

Results obtained for the helical hairpin are visualized in Figure 1A, which provides a comparison of two distributions (T and O), revealing fragments where the observed distribution clearly diverges from the theoretical model. According to the fuzzy oil drop model fragments characterized by a local excess of hydrophobicity may play a role in the complexation of external structures which also expose excess hydrophobicity on their surface (in the case of the presented structure, complexation involves the micelle). Judging by *RD* and correlation coefficients, this fragment of the ASyn chain lacks a well-defined hydrophobic core (refer to Table S1—Note that all tables are available in Supplementary Materials, indexed by “S” together with the table number)—Although eliminating residues which exhibit deviations from the theoretical distribution produces a fragment whose *RD* value is below 0.5. Figure 1B identifies locations where interaction with external molecules is likely to occur.

The complete micelle-bound form of Asyn (1XQ8) can be characterized (on the basis of fuzzy oil drop parameters) as lacking a hydrophobic core, which is a direct consequence of its notable lack of a tertiary conformation. If, however, we limit our analysis to the helical hairpin (1–95), we may observe a tendency for hydrophobic residues to congregate in the central part of the molecule and for hydrophilic residues to migrate to its surface. Eliminating residues which exhibit the greatest difference between T_i_ and O_i_ reveals fragments contributing to the formation of a centralized core. These fragments (highlighted in Figure 1A) are likely primed for interaction with the hydrophobic surface of the target molecule, and their identification bases on comparing the theoretical (T) and observed (O) distributions of hydrophobicity (Figure 1B).

Elimination of residues highlighted in Figure 1A,B reduces the value of *RD* to below 0.5, indicating good agreement with the Gaussian distribution.

The status of the 1–95 fragment of micelle-bound ASyn (Table S1) appears to indicate the lack of a centralized core; however, eliminating residues highlighted in Figure 1 as representing a local deviation from T (in the form of excess or insufficient hydrophobicity) produces a structure which is a good match for the centralized model.

Given the extensive literature devoted to the properties of specific ASyn fragments, Table S1 also lists the status of these fragments—This information will come in handy when discussing their conformation in the amyloid form of ASyn. Additionally, we have computed the status of certain repetitive fragments and other fragments which appear in previously published studies.

Repetitive fragments are generally found to deviate from the theoretical distribution—In fact, values listed in Table S1 indicate that some of these fragments exhibit amyloid-like properties. The status of fragments which adopt beta folds in the amyloid is also quite similar to an amyloid structure, particularly in the case of the 70–78 fragment. Helical fragments (in micelle-bound ASyn) exhibit similar properties to those identified in amyloid structures (high *RD* and significant correlation bias, with negative values of HvT and TvO and strongly positive values of HvO).

The 25–35 fragment, implicated in the onset of Parkinson’s disease [6], exhibits amyloid-like properties in micelle-bound ASyn, as do two other fragments—1–25 and 26–98 (which, according to Reference [5], are responsible for anchoring to the lipid bilayer). Other fragments have been singled out, due to their status in the amyloid protein (see the “Amyloid” tag in Table S1), as discussed further below.

### 2.3. Structure of the Amyloid Form of ASyn (2N0A)

**Figure 2:**
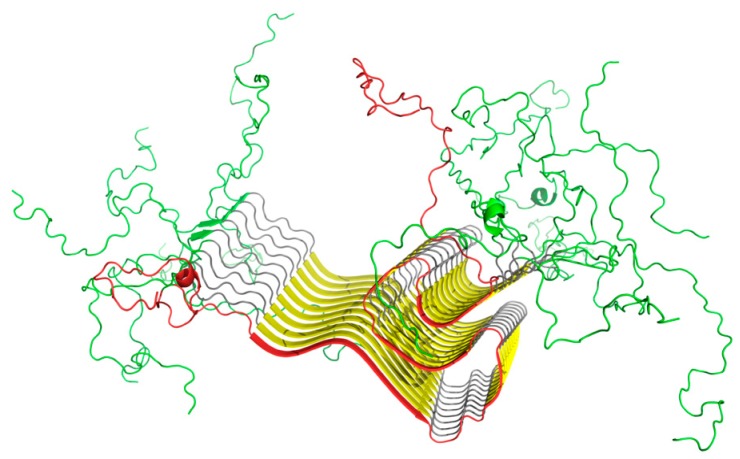
3D structure of the amyloid form of ASyn, with a clearly distinguished amyloid-like section (30–100). Chain A has been marked red, while random coil fragments (1–29 and 101–140) are shown in green.

This part of our analysis focuses on the amyloid form of ASyn, listed in PDB under ID 2N0A and visualized in Figure 2.

**Figure 3:**
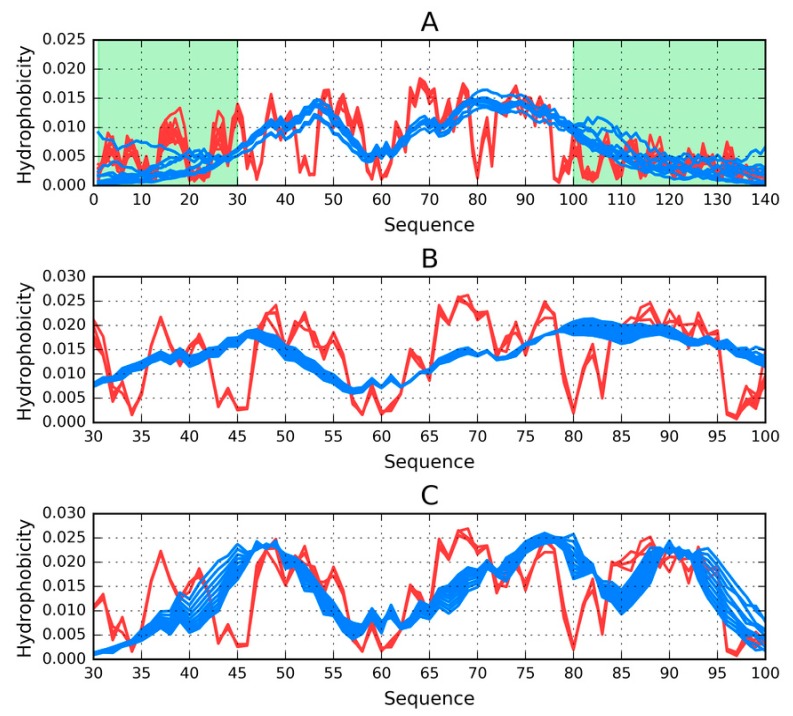
Theoretical (T, blue) and observed (O, red) hydrophobicity density distribution profiles for the ASyn amyloid (2N0A). Each chart shows two profiles for every chain from the complex (10 in total); (**A**)—Calculations performed for the entire complex (1–140). Random coil fragments (1–29, 101–140) are highlighted in green; (**B**)—Calculations performed for the amyloid fragment (30–100) treated as part of the complex; (**C**)—Calculations performed for the amyloid fragment (30–100) treated as an individual molecule.

Figure 3A presents T and O distributions for the complete amyloid form of Asyn. The theoretical distribution contains two distinct hydrophobicity maxima; however, the observed distribution does not replicate this pattern and instead exposes numerous local maxima, including in the N- and C-terminal fragments, both of which are disordered (Figure 3). The presence of such local maxima in these fragments is likely due to close-range interactions with neighbors belonging to the same chain. This phenomenon differs from the emergence of alternating “bands” of hydrophobicity, although it might suggest that these terminal fragments may also be susceptible to producing amyloid-like structures. This brings up the following question—Why does the fibrillar conformation of ASyn not extend to its N-terminal fragment?

**Figure 4:**
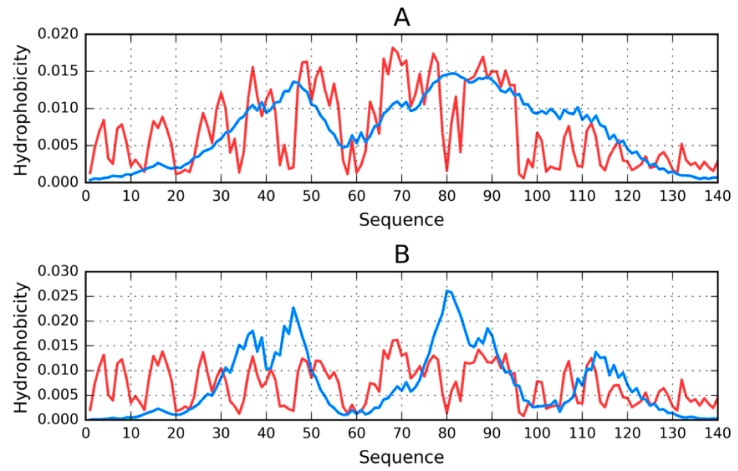
Theoretical (T, blue) and observed (O, red) hydrophobicity density distribution profiles for chain E (central) from the ASyn amyloid (2N0A); (**A**)—Treated as part of the complex; (**B**)—Treated as a standalone structure.

When the fibrillar fragment is treated as part of a larger complex (Figure 3B) or considered on its own (by restricting the 3D Gaussian capsule to that fragment alone–see Figure 3C), the resulting distribution of hydrophobicity is invariably found to contain numerous local maxima, similar to those presented in Figure 3A,B In order to avoid potentially misleading interpretations of the presence of alternating hydrophobicity bands in the N- and C-terminal areas, the presented computations take into account only cross-chain interactions (Figure 4). Here, local maxima resulting from the fibrillar conformation of the 30–100 fragment are clearly visible.

The values listed in Table S2 reveal the unique status of the amyloid, which–when considered in its entirety—Exhibits a distribution of hydrophobicity consistent with the presence of a central hydrophobic core (this observation holds even when the analysis is restricted to cross-chain interactions). The properties of the fibrillar section are also quite well aligned with the monocentric core model. Indeed, eliminating residues 80–83 brings the value of *RD* down to less than 0.5.

Table S2 provides quantitative information for the presented complex and its constituent parts, as discussed above.

Assessment of the status of residues 1–30 and 100–140 falls out of the scope of the fuzzy oil drop model, due to the lack of cross-chain interactions. In this specific case, the observed distribution is the result of mutual interactions between residues belonging to each individual chain, and does not reflect the role performed by these fragments in the analyzed complex.

There is good agreement between the theoretical and observed distribution of hydrophobicity in the fibrillar fragment, which may be regarded as surprising. On the basis of the fuzzy oil drop model, we can propose the following explanation: The model assumes that a globular protein emerges as a result of interactions between its residues and the aqueous solvent. This process causes migration of hydrophobic residues towards the center of the emerging globule, along with exposure of hydrophobic residues on its surface. If, however, the properties of the environment are altered so that the environment is not capable of acting upon the protein chain, forces associated with the intrinsic hydrophobicity of individual residues take over. By “properties of the environment” we refer to a continuous external force field generated by a specific arrangement of water molecules surrounding the protein (note, however, that the structural properties of water in its liquid phase remain poorly understood).

In its micelle-bound form (1XQ8) ASyn is relatively well aligned with the predictions of the fuzzy oil drop model; however, it can only retain stability in the presence of a “permanent chaperone”. In vivo, the role of this chaperone typically falls to nerve cells, while the structure listed in PDB is stabilized by a micelle. When deprived of contact with its target molecule, ASyn undergoes significant conformational changes, leading to the emergence of an amyloid structure.

It should also be noted that the fibrillar part of ASyn is surrounded not by water, but by a buffer zone occupied by the randomly coiled terminal fragments, as shown in Table S3 and Figure 2.

### 2.4. Models Generated by Specialized Software

Our discussion of models produced by specialized software toolkits begins with a study of the structure of individual chains. We singled out the E chain for in-depth analysis, due to its central location in the sample fibril, which makes it the best available match for a fibril of arbitrary length. For the purposes of our analysis, the chain was treated as part of the larger complex, as well as a standalone structure. In the former case, we assessed its alignment with the overall distribution of hydrophobicity in the amyloid fibril, whereas, in the latter case our focus was on alignment with the theoretical distribution of hydrophobicity (T) given by the 3D Gaussian.

When the E chain is considered as part of the complex, its distribution approximates that which is evident in the complex as a whole (Figure 4A). On the other hand, calculating the distribution of hydrophobicity for the standalone chain reveals—In addition to similar gaps between local peaks at 43–46 and 79–83—An additional gap at 43–46. It should be noted that all such gaps occur within repetitive fragments and correspond to relatively high polarity, revealing strong discordance vs. the theoretical distribution. In addition, the 66–71 fragment (VGGAVV) represents a local excess of hydrophobicity.

The above properties, illustrated in Figure 4B, provide a set of references for the analysis of models generated by our 3D protein structure modeling software.

### 2.5. Analysis of Models for the 1–140 Fragment of ASyn

In order to facilitate comparative analysis, we computed 3D structural models for the entire sequence of ASyn (1–140). When comparing results, we focus on parameters which indicate alignment (or lack thereof) with the globular hydrophobic core model, revealing that none of the obtained models carries the properties of a spherical micelle.

Table S4 summarizes the results of structure prediction studies carried out for the ASyn (1–140) polypeptide. It appears that this polypeptide is incapable of adopting a distribution of hydrophobicity which would correspond to a spherical micelle, even though some models (mostly those produced by FOD) suggest a globular conformation. In this case, high values of correlation coefficients reveal an amyloid-like pattern with a strong bias towards intrinsic hydrophobicity.

I-Tasser produced a single model with balanced values of TvO and HvO; however, for this model, the value of *RD* (T-O-R) remains high. Robetta generated five distinct models, all of which are characterized by *RD* > 0.5 (except for T-O-H), with balanced values of TvO and HvO. The FOD toolkit produced 500 distinct models. From among these, models with the highest and lowest values of *RD* were selected for further analysis.

**Figure 5:**
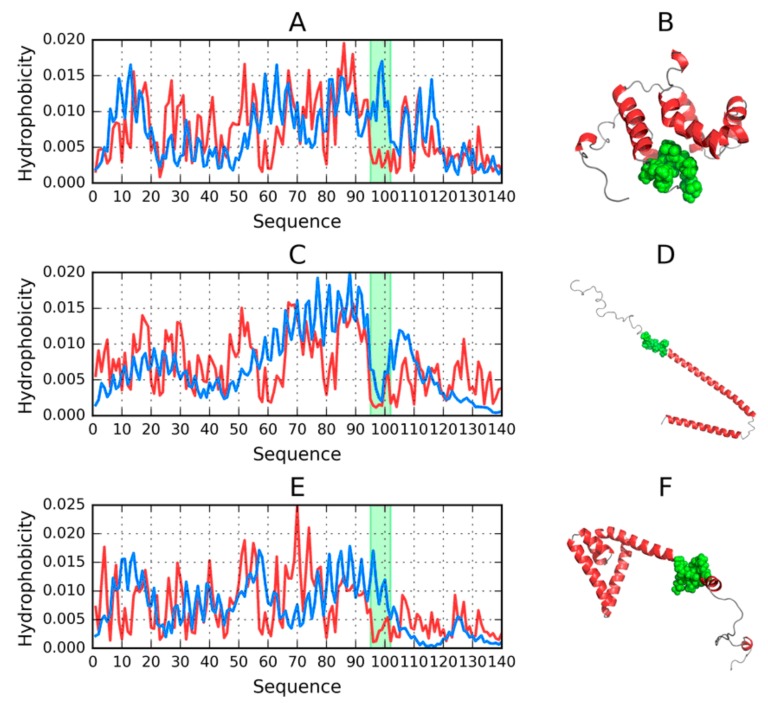
3D visualizations along with theoretical (T, blue) and observed (O, red) hydrophobicity density distribution profiles for models of the 1–140 fragment produced by each software package and characterized by the lowest RD. The 95–102 fragment is highlighted in green; (**A**,**B**)—FOD_1_(202); (**C**,**D**)—ITASSER_1_(1); (**E**,**F**)—ROBETTA_1_(2).

Figure 5 reveals strong agreement between T and O in the N- and C-terminal fragments. Major differences concern the expected hydrophobic core, which, in the I-Tasser model, appears to begin at residue 50, while in the PDB structure its beginning is located at residue 70. The Robetta model reveals greater involvement of the N-terminal fragment in shaping the protein’s hydrophobic core, which is not evident in the PDB structure. Neither of these structures can be characterized as globular—The reason behind generating various models is to determine whether the ASyn sequence is at all capable of producing a globular fold. Such structures are (for obvious reasons) produced by FOD simulations; however, their status does not correspond to the properties of spherical micelles (in particular, *RD* (T-O-R) is greater than 0.5). Further analysis of distribution profiles highlights the causes of this phenomenon (Figure 3).

Eliminating the 95–102 fragment yields the desired value of *RD* (T-O-R), producing a structure which is quite similar to the target. Notably, FOD computations generate a large variety of models (500 in total), and it should be noted that FOD generally favors the formation of a hydrophobic core resembling a spherical micelle. We may, therefore, conclude that the ASyn sequence is, indeed, incapable of producing a soluble protein. It is also worth noting that the corresponding fragment of the micelle-bound ASyn structure (derived from PDB) exhibits similar discordance. This fragment has been highlighted in Figure 5A (1–140) and Figure 5B (1–140), as well as in Figure 1, and appears to be the causative factor determining the presented conformational properties of ASyn. It retains strong discordance in Robetta models, while I-Tasser deals with it by exposing it on the surface.

### 2.6. Structure of the N-Terminal Fragment in FOD, I-Tasser and Robetta Models (1–30 aa)

In the ASyn amyloid form listed in PDB (2N0A) the 1–30 fragment does not belong to the fibril. Instead, it is characterized as a random coil, with a disordered, nonrepetitive structure. It is, therefore, interesting to speculate whether, under favorable conditions, this fragment may produce a globular fold. To address this issue, we carried out using FOD, I-Tasser and Robetta.

Table S5 provides a summary of results, revealing strong variability of models produced for the N-terminal fragment of the ASyn chain. This fragment was selected for analysis, due to its disordered structure in the ASyn amyloid (2N0A). Results can be described as highly variable. The FOD model produced a distribution consistent with the theoretical model, suggesting the presence of a globular form with a centrally located hydrophobic core. I-Tasser models also hint at the possibility of generating this type of structure, although they provide two alternative structures. Regarding Robetta, all of its models diverge from the theoretical distribution with a clear preference for helical folds dominating the entire fragment. Helical folds are also evident in I-Tasser and FOD models, although in their case the presence of twists results in globular conformations (particularly in FOD models). Both FOD and I-Tasser structures are dominated by the TvO correlation coefficient, while for other models, the HvO coefficient prevails.

**Figure 6:**
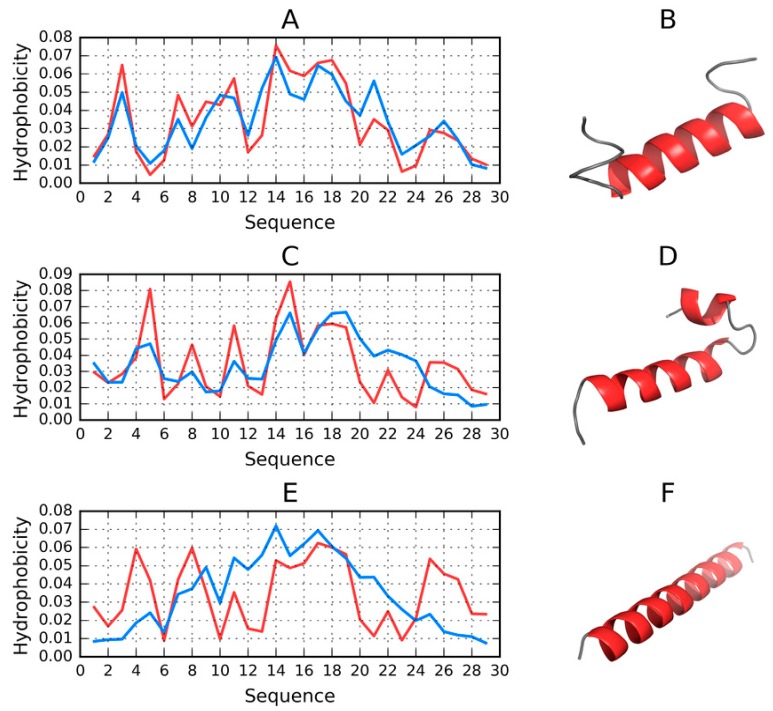
3D visualizations along with theoretical (T, blue) and observed (O, red) hydrophobicity density distribution profiles for models involving the 1–30 fragment, produced by each software package and characterized by the lowest RD. (**A**,**B**)—FOD_1_(053); (**C**,**D**)—ITASSER_1_(3); (**E**,**F**)—ROBETTA_1_(4).

Figure 6 provides a visualization of profiles and 3D structures of selected models.

One conclusion which can be drawn from the analysis of the 1–30 sequence is that it admits a globular conformation with a centralized hydrophobic core (in the case of the FOD model, some structures selected from among the 500 output models exhibit this status).

### 2.7. Structure of the 30–100 Fragment of the ASyn Polypeptide According to FOD, I-Tasser and Robetta

This fragment should be regarded as particularly important, since it represents the fibrillar core of the ASyn amyloid. Consequently, it is interesting to speculate whether it can produce a spherical micelle.

**Figure 7:**
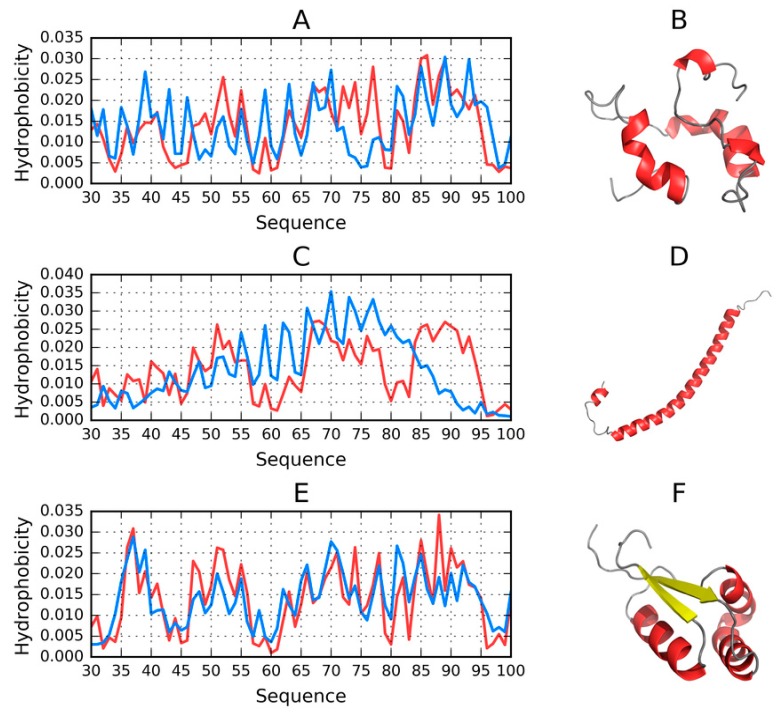
3D visualizations along with theoretical (T, blue) and observed (O, red) hydrophobicity density distribution profiles for models of the 30–100 fragment, produced by each software package and characterized by the lowest RD. (**A**,**B**)—FOD_1_(289); (**C**,**D**)—ITASSER_1_(1); (**E**,**F**)—ROBETTA_1_(2).

The summary presented in Table S6 suggests that only two models approximate the spherical micelle. Their status is also visualized in Figure 7.

FOD_1 satisfies *RD* < 0.5, although its 72–77 fragment deviates from the theoretical distribution of hydrophobicity (by being overly hydrophobic). Eliminating this fragment results in much better alignment between both profiles, with *RD* (*T-O-R*) = 0.394.

Similarly, the structure of the I-Tasser model exhibits a local excess of hydrophobicity in its 79–94 fragment. Eliminating this fragment brings RD down to 0.468, suggesting good alignment with the spherical micelle pattern for the majority of the proposed structure.

Robetta also generates a conformation largely consistent with the spherical pattern. It is, in fact, the only toolkit which predicts the presence of beta folds in the analyzed chains (in models 1 and 2). The remaining models (3–5) are purely helical. The model visualized in Figure 7 represents the best match for the spherical pattern from among all analyzed models.

Summarizing our study of the conformational capabilities of the 30–100 fragment of ASyn, we need to note that this sequence is capable of adopting conformations which correspond to the spherical micelle pattern—Even though the vast majority of proposed models represent other patterns.

### 2.8. Comparative Analysis of Models Obtained for the 100–140 Fragment of ASyn

The C-terminal fragment of the ASyn amyloid (100–140) (2N0A) has been experimentally determined to adopt a highly disordered conformation, usually described as a random coil, with no obvious secondary folds. It is, therefore, interesting to speculate whether this fragment is at all capable of achieving an orderly secondary structure. In order to answer this question, we calculated a series of models using FOD, I-Tasser and Robetta.

Summarizing the parameters listed in Table S7 we may conclude that the C-terminal fragment (100–140)—which becomes a random coil in the protein’s micelle-bound form—May adopt a globular conformation. From among the models produced by Robetta, only two structures do not conform to this pattern (*RD* (*T-O-R*) ≥ 0.5).

Most of the generated models are dominated by short helices and random coil fragments. Again, Robetta provides an exception, with a notable presence of beta folds in two models for which *RD* < 0.5.

The fact that the C-terminal fragment readily adopts globular conformations is all the more surprising given its high polarity and sparsity of hydrophobic residues.

All structures which satisfy *RD* < 0.5 are also characterized by balanced values of correlation coefficients, with no significant bias.

**Figure 8:**
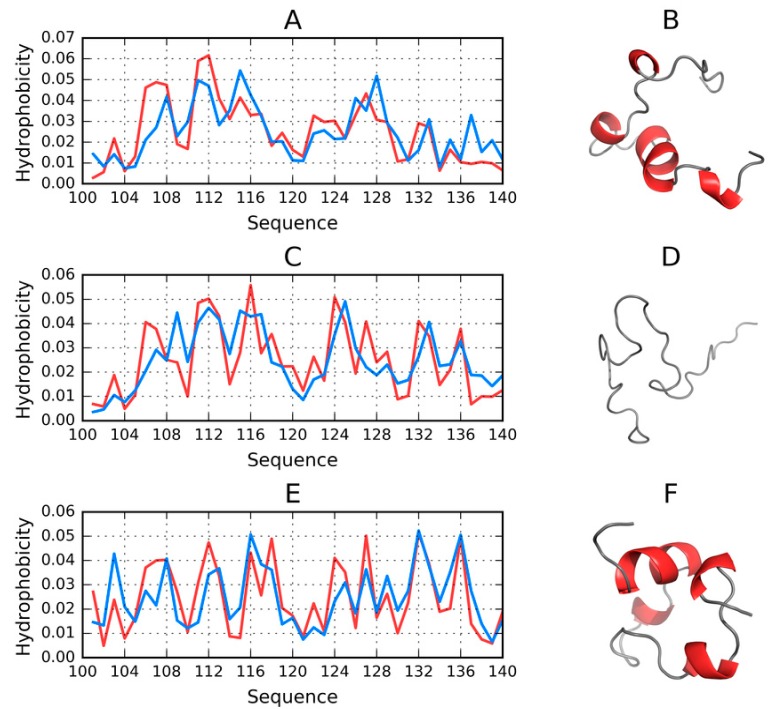
3D visualizations, theoretical (T, blue) and observed (O, red) hydrophobicity density distribution profiles for models of 100–140 fragment, produced by each software package and characterized by the lowest RD. (**A**,**B**)—FOD_1_(002); (**C**,**D**)—ITASSER_1_(5); (**E**,**F**)—ROBETTA_1_(1).

In conclusion, it appears that the 100–140 fragment of ASyn is capable of adopting a globular conformation with a prominent hydrophobic core, approximating a spherical micelle. Of course, this does not imply that the fragment retains this capability in the context of a larger structure—As evidenced by the lack of globular models for the complete chain of ASyn (1–140) (Figure 8).

### 2.9. Effect of Mutations on Amyloid Transformation

**Figure 9:**
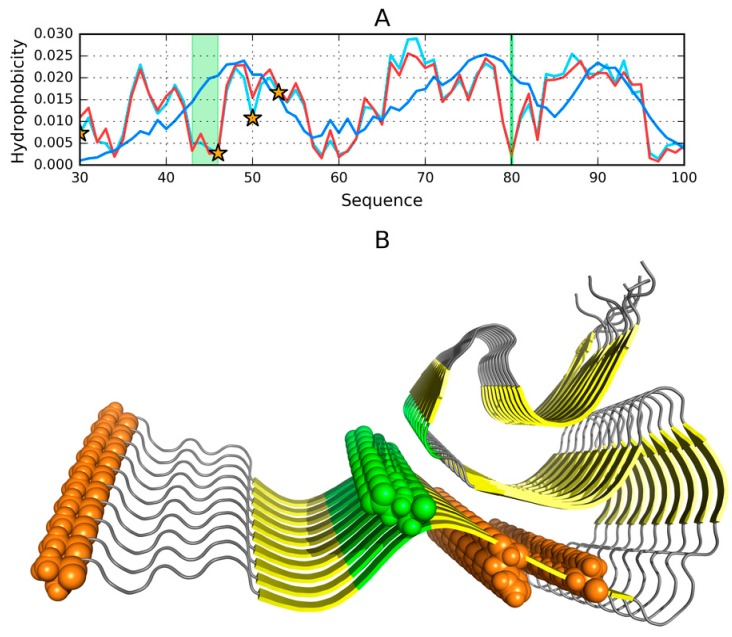
Presentation of the fibrillar part (30–100) of ASyn (2N0A); (**A**)—Theoretical (T, blue) and observed (O, red) hydrophobicity density distribution profiles for chain E. The teal chart represents the observed profile for the mutated sequence; (**B**)—3D visualization; Orange stars in Figure 9A, and orange/green spheres in Figure 9B correspond to loci of mutations which lower intrinsic hydrophobicity: A30P, E46K, H50Q and A53T. Green fragments correspond to residues No. 43–46 and 80, which–when eliminated from FOD computations—Produce a distribution consistent with the theoretical model (*RD* lowered from 0.506 to 0.490).

The effect of experimentally observed mutations on the amyloid transformation of ASyn may be studied on the basis of the fuzzy oil drop model, which provides a way to predict local conformational changes resulting from changes in the underlying distribution of hydrophobicity. This is visualized in Figure 9, which highlights several mutation loci (A53T, E46K, A30P and H50Q) [33]. In all of these cases, the intrinsic hydrophobicity of the substituent residue is lower than that of the original residue (according to any acknowledged intrinsic hydrophobicity scale).

The diagrams are shown in Figure 9A, when confronted with the corresponding locations indicated in Figure 9B, reveal the specificity of the mutated residues.

The effect of A30P is described as neutral with regard to the protein’s susceptibility to undergo amyloid transformation. From the point of view of the fuzzy oil drop model, residue 30, which is located in the fibril’s outer layer, may admit a local reduction in hydrophobicity [34]. Residues 46, 50 and 53 are also found in the outer layer. Reducing their hydrophobicity may, therefore, mediate entropically favorable interactions with the aqueous solvent, stabilizing the ribbonlike micelle.

Analysis of Figure 9 suggests favorable conditions for the emergence of a ribbonlike micelle, with an internal hydrophobic core propagating along the fibril’s axis.

### 2.10. Status of Selected Fragments Identified in Other Publications as Linked to Amyloid Transformation in Parkinson’s Disease

The selected fragments are implicated in the onset of Parkinson’s disease [5]. The status of this fragment in the micelle-bound form of ASyn (1XQ8) is clearly amyloid-like, whereas, in the true amyloid (2N0A) it adopts a peculiar conformation, while retaining *RD* < 0.5. The fragment itself is located at the junction of the random coil and fibrillar sections. We have previously demonstrated that the 1–30 fragment may fold as a globule, while the 30–100 fragment lacks this capability. Thus, the 25–35 fragment may potentially play the role of an amyloid seed.

Fragments tagged as amyloid-like in Tables S2 and S3 can be identified by searching for a distribution of hydrophobicity which stands in opposition to the globular pattern. Their status also mostly reflects conditions which are likely to be encountered in amyloids. While investigating the causes of amyloid transformation, it is useful to refer to the sequences of such fragments. This work is currently ongoing and will be the subject of a separate publication.

Table S8 provides a summary of results obtained for fragments which other authors identify as amyloidogenic. It is clear that the structural properties of the generated models vary widely. Values listed in boldface correspond to classic amyloid-like conditions, which are understood as a combination of high values of RD, low (potentially negative) values of HvT and TvO and strongly positive values of HvO. Taken together, these parameters indicate that the conformation of the given fragment is driven by the intrinsic properties of its component residues.

The aim of this presentation is to highlight the structural variability of amyloidogenic fragments, while establishing that they generally diverge from the centralized hydrophobic core model. Given the latter, it should come as no surprise that these fragments are capable of producing an alternative (fibrillar) structural pattern.

## 3. Discussion

Amyloid nucleation of ASyn is often linked to the 68–82 fragment, which may initiate the early assembly of ASyn [34] Assessment of the role which this fragment plays in the stabilization of the ASyn amyloid is facilitated by the characteristics of the 61–95 fragment. Of note is the hydrophobic seed emerging in the 68–82 area, consistent with that fragment’s status, shown in Figure 9A, and indicating an atypical, amyloid-like distribution of hydrophobicity.

The status of the 1–30 and 101–140 fragments remains puzzling. The presence of identical sequences in adjacent chains may create favorable conditions for complexation and generation of variably hydrophobic bands, similar to what can be observed in the central section (30–100). The 1–30 fragment was only capable of adopting a centric fold in FOD-based calculations, while Robetta did not produce any corresponding globular structures. Regarding the 30–100 fragment, only two of the obtained models predict a status consistent with a soluble protein, while in the case of the 100–140 fragment most models indicate the capability for producing a globular form with a coherent hydrophobic core. Considering the relative hydrophilicity of participating residues, this observation suggests that the terminal fragment “seeks” an alternative (non-fibrillar) conformation and may attain it under certain conditions.

Intensive studies concerning the ability of ASyn and Tau to undergo amyloid transformation often link their properties to the presence of intrinsically disordered fragments and polyperolin-like forms (Ramachandran map), regarded as likely amyloid seeds [35]. In general, the tertiary and quaternary structure of proteins stems from cooperative interaction between individual residues and the aqueous solvent, whose presence is an essential prerequisite of life. This cooperation typically results in the formation of a hydrophobic core overlaid by hydrophilic residues, roughly accordant with the 3D Gaussian distribution of hydrophobicity. Analysis of individual protein domains confirms that almost invariably comply with this pattern [36], while any local discordances are usually associated with biological function [32]. Cooperative tendencies, which produce a 3D Gaussian distribution of hydrophobicity in a globular protein, may instead result in a 2D Gaussian distribution when an amyloid unit chain is considered [31].

The comparative analysis of ASyn amyloids and tau proteins presented in Reference [37] is also of interest to us. The FOD model underscores the role of the environment, and of the solvent in particular. The role of membranes as a factor promoting amyloidogenesis is discussed in Reference [38,39].

The link between mutations and amyloidogenesis is undisputed [40]. Nevertheless, the environment—Especially an altered environment (in terms of pH and ionic strength)—Is frequently indicated as a factor which promotes the amyloid transformation of ASyn, including its mutated forms (A30P, E46K, G51D and A53T) in α-synuclein fibrils [41].

Analogies with prions may provide a lead in the search for the causes of amyloid transformation [42,43].

The search for new drugs focuses on dopamine (Dopa), amphotericin-B (Amph), epigallocatechingallate (EGCG), and quinacrinedihydrochloride (Quin) as factors affecting the oligomerization of ASyn [44,45].

Co-immunoprecipitation is also considered as a potential therapeutic technique [46]. Selection of the 30–100 fragment based on analysis of the ASyn amyloid structure remains consistent with the outcome of experimental studies which single out the NAC fragment (60–100) as particularly prone to amyloid transformation [47] An open question is why the fragments at 1–30 and 100–140, despite repeating the same sequence in all unit chains, do not participate in propagation of the fibril. Based on the analysis presented in this paper, we suggest that their tendency to generate a globular structure with a prominent hydrophobic core may preclude fibrillization. Strong hydrophilicity of residues comprising the 100–140 fragment enables penetration of water; however, the presence of the adjacent fibril (at 30–100) prevents the formation of a globule. Consequently, the random coil remains the only possible alternative. We should also note the lack of fibrillar properties in the 1–30 fragment, which, given its structural properties, might be suspected as being capable of producing a fibril. The ongoing analysis focuses on the properties of sequences which comprise known amyloids, as well as each fragment of the ASyn polypeptide (with a publication currently in preparation).

The fuzzy oil drop model describes and expects a spherical, centralized hydrophobic core. ASyn proves that a centralized hydrophobic core may also be present in a fibrillar structure. Summarizing the presented results, we may propose that when the chain is unable to “resolve” to a spherical micelle, with all of its hydrophobic residues isolated in the central part and all hydrophilic residues exposed on the surface, it instead adopts a ribbon-like micellar conformation. This structure is characterized by advantageous entropic effects, including the isolation of hydrophobic residues within a central (in the sense of a horizontal cross-section) band stretching along the fibril’s axis. The ASyn amyloid is, in many respects, unique, especially when compared to Aβ(1–42) amyloids. As a result, its analysis reveals interesting aspects of the amyloid transformation process. For example, it turns out that the presence of long disordered N- and C-terminal fragments promote isolation of the central fibril, which contains the aforementioned bandlike hydrophobic core. Additionally, we reveal a common mechanism, observed in both globular and fibrillar structures, where the polypeptide chain attempts to isolate its hydrophobic residues from direct contact with the solvent. We are also currently involved in performing a comparative analysis of various amyloid structures listed in PDB, in search for general amyloid formation mechanisms which apply regardless of specific sequential properties [31].

As it is shown in this paper, the comparative analysis reveals no preference for globular forms for the amino acids sequence as it appears in ASyn. The models represent the structures expressed by *RD* > 0.5 (see Tables in Supplementary Materials). It suggests that the sequence specificity directs the folding process toward other than globular forms. It is also observed in other amyloids, the structure of which is available in Protein Data Bank [39]. The generalization of rules directing the folding process toward ribbon-like micelles is the object of currently conducted analysis.

## 4. Materials and Methods

### 4.1. Data

The structure of the amyloid form of ASyn (PDB ID: 2N0A) consists of 10 chains, where—Unlike other amyloids listed in PDB [10,11,12,13,14,48]—Only a portion of each chain adopts a fibrillar form (in contrast, the entire chains of Aβ (1–42) amyloids participate in the formation of a fibril. In the case of ASyn, both the N-terminal fragment (1–30) and the C-terminal fragment (100–140) are random coils, while the 31–100 fragment can be characterized as a typical amyloid fibril in all 10 chains. This structural diversity, along with its underlying causes, make ASyn an interesting study subject.

Based on the above observations and on the published, experimentally determined structure of the ASyn amyloid (PDB ID: 2N0A), we distinguish three fragments: 1–30 (random coil), 31–100 (fibril) and 101–140 (random coil). Notably, PDBSUM [49] identifies the following beta folds in ASyn: 38–55, 60–67, 70–78, 81–84 and 88–97. PDB also provides structural information for a single chain of ASyn (PDB ID: 1XQ8). This micelle-bound form comprises two helical fragments (2–38 and 44–93) linked by a hairpin with a tight bend at 39–43. The C-terminal fragment (identified as 94–140) is, again, described as a random coil. We will use this structure as the reference for the amyloid form of ASyn, as well as for models produced by our software.

### 4.2. Obtaining Alternative ASyn Polypeptide Models

In order to determine whether ASyn is capable of adopting other conformations than those previously listed, we have carried out using I-Tasser (University of Michigan, Ann Arbor, MI, USA) [18] and (Robetta Department of Biochemistry, University of Washington, Seattle, WA, USA) [17]—Specialized protein structure prediction toolkits, both of which rank among the best in the CASP (Critical Assessment of Structure Prediction) challenge [19]. They both operate upon independently defined force fields and apply different algorithms (e.g., for energy minimization)—Thus, they can be expected to produce alternative structural forms for the ASyn sequence. In line with the CASP challenge rules, Roberta was used to producing five models, while I-Tasser generated between 1 and 5 models depending on the length of the input chain.

All calculations were carried out using online servers [29,30]. In addition, a separate calculation was performed using a software toolkit based on the FOD model (Jagiellonian University—Medical College, Krakow, Poland) [20,21,22], which, in addition to internal free energy optimization, also optimizes interactions with an external force field representing the aqueous solvent. This force field is mathematically defined as a 3D Gaussian and its presence results in internalization of hydrophobic residues, with the attendant exposure of hydrophilic residues on the protein surface. The presented calculations were conducted using the Gromacs (Rijksuniversiteit Groningen, Groningen, Netherlands) package [50], with optimization of internal and external force fields carried out in an interleaved fashion. Output models were ranked by their values of the *RD* (Relative Distance—See the section titled “Fuzzy oil drop model—Protein folding with the preferential generation of a hydrophobic core”), allowing us to select models which most closely approximate a spherical micelle.

According to the fuzzy oil drop model, the presence of the aqueous solvent favors the generation of globular structures containing hydrophobic cores—Therefore, the model can be used to determine whether a given polypeptide is capable of achieving a globular confirmation. FOD application requires the user to provide an input (starting) structure. In our case, we used the structure of an individual polypeptide belonging to the presented fibril (2N0A). The goal of these calculations was to identify alternative folding patterns which may manifest themselves under altered environmental conditions. Protein structure prediction was carried out for the entire ASyn chain (1–140), as well as for the previously identified fragments (1–30, 30–100 and 100–140).

FOD based computations were performed at the Academic Computing Centre CYFRONET AGH using resources provided by the PL-Grid (University of Science and Technology, Kraków, Poland) infrastructure [51].

### 4.3. Fuzzy Oil Drop Model—Protein Folding with Preferential Generation of a Hydrophobic Core

The base model has been thoroughly described in numerous publications [20,21,22]. At its core rests the assumption that a globular protein contains a hydrophobic core, which (in its “idealized” or “theoretical” version) can be mathematically modeled as a 3D Gaussian superimposed upon the protein body. Thus, each effective atom (averaged-out positions of all atoms comprising a single residue) has a theoretical hydrophobicity value (T_i_) given by the Gaussian. In addition, each residue also carries the so-called observed hydrophobicity (O_i_), which is dependent on its hydrophobic interactions with adjacent residues. Such interactions depend on the separation between residues and on their intrinsic hydrophobicity, which is defined according to the scale proposed in Reference [52].

The following algorithm is applied to compute the theoretical and observed hydrophobicity in a protein molecule:

The molecule is oriented in such a way that A—Its geometric center coincides with the origin of the coordinate system; B—The longest axis of the molecule coincides with the *X* axis; C—The line connecting the two most distal atoms (projected on the YX plane) corresponds to the *Y* axis.

For each axis the greatest separation between any two atoms is computed and subsequently multiplied by 6, yielding values of σ_x_ σ_y_ σ_z_, respectively.

For each residue, the position of its effective atom is calculated by averaging out the positions of all atoms which belong to the given residue.

The value of the 3D Gaussian at the point corresponding to the effective atom is taken as theoretical hydrophobicity (again, for the given residue). This can be mathematically expressed by the following formula:

(1) $$\tilde{H}t_{j}=\frac{1}{\tilde{H}t_{sum}}\exp\left(\frac{-{\left(x_{j}-\bar{x}\right)}^{2}}{2\sigma_{x}^{2}}\right)\exp\left(\frac{-{\left(y_{j}-\bar{y}\right)}^{2}}{2\sigma_{y}^{2}}\right)\exp\left(\frac{-{\left(z_{j}-\bar{z}\right)}^{2}}{2\sigma_{z}^{2}}\right)$$

where $\tilde{H}t_{j}$ describes the theoretical hydrophobic density (hence, the *t* index) at point *j*.

Hydrophobic interactions between residues are calculated based on Levitt’s formula [27], which depends on intrinsic hydrophobicity (according to some predefined scale—See Reference [22]) and on the separation between interacting residues:

(2) $$\tilde{H}o_{j}=\frac{1}{\tilde{H}o_{sum}}\sum_{i=1}^{N}\left(H_{i}^{r}+H_{j}^{r}\right)\left\{\begin{array}{l} \left[1-\frac{1}{2}\left(7{\left(\frac{r_{ij}}{c}\right)}^{2}-9{\left(\frac{r_{ij}}{c}\right)}^{4}+5{\left(\frac{r_{ij}}{c}\right)}^{6}-{\left(\frac{r_{ij}}{c}\right)}^{8}\right)\right]\\ 0\;\mathrm{for}\;r_{ij}\leq c\; \end{array}\right.\;\mathrm{for}\;$$

where *N* is the number of amino acids in the protein, $\tilde{H}$${}_{i}^{r}$ expresses the hydrophobic parameter of the *i*-th residue, while *r_ij_* expresses the distance between two interacting residues (*j*-th “effective side chain” and *i*-th “effective side chain”). C is the cutoff distance—Assumed equal to 9 Å.

All values of Ti and Oi are subjected to normalization to ensure that they add up to 1.

Plotting the values of Ti and Oi for successive amino acids in the polypeptide chain reveals differences between both distributions, as well as fragments where they remain closely aligned.

In order to quantitatively express the differences, divergence entropy [28] is computed according to the following formula:

(3) $$D_{KL}\left(p\left|p^{0}\right.\right)=\sum_{i=1}^{N}p_{i}\log_{2}\left(p_{i}/p_{i}^{0}\right)$$

where $p_{i}$—Observed probability, $p_{i}^{0}$—Reference probability, N—Number of residues in the chain

Given that Equation (3) yields a measure of entropy, the resulting value cannot be interpreted on its own. To make meaningful observations, another reference distribution is required. In our case, this second “boundary” distribution is called R and denotes a case where all residues carry identical hydrophobicity (which is similar to the distribution of electrostatic interactions in many proteins [52]). Accordingly, the value of Ri for each residue is 1/N, N being the number of residues in the chain.

Comparing D_KL_ for the O-T relation and for the O-R relation tells us whether the observed distribution more closely approximates the 3D Gaussian form (O-T < O-R) or the uniform pattern (*O-T* > *O-R*). In order to avoid having to deal with two distinct values, we compress them into a single parameter, referred to as Relative Distance, as follows:

(4) $$RD=\frac{O\left|T\right.}{O\left|T+O\left|R\right.\right.}$$

As noted above, *RD* < 0.5 is interpreted as accordance between O and T, indicating the presence of a hydrophobic core. In all other cases, we assume that the protein body lacks a clearly defined core.

The concept of *RD*—Relative Distance also enables us to assess the status of a specific fragment of the polypeptide chain (following normalization of Ti, Oi and Ri values). Eliminating residues for which Ti strongly deviates from Oi furthermore tells us which part of the polypeptide chain gives rise to the hydrophobic core.

The presented analysis may also be carried out for a case where the uniform distribution ^®^ is replaced by a distribution reflecting the intrinsic hydrophobicity of each residue (denoted H). In this case, *RD* < 0.5 indicates the presence of a hydrophobic core, while *RD* > 0.5 suggests that the observed conformation of the protein is determined mostly by the intrinsic (“selfish”) properties of its constituent residues, which override the previously described “cooperative” interactions with the solvent.

**Figure 10:**
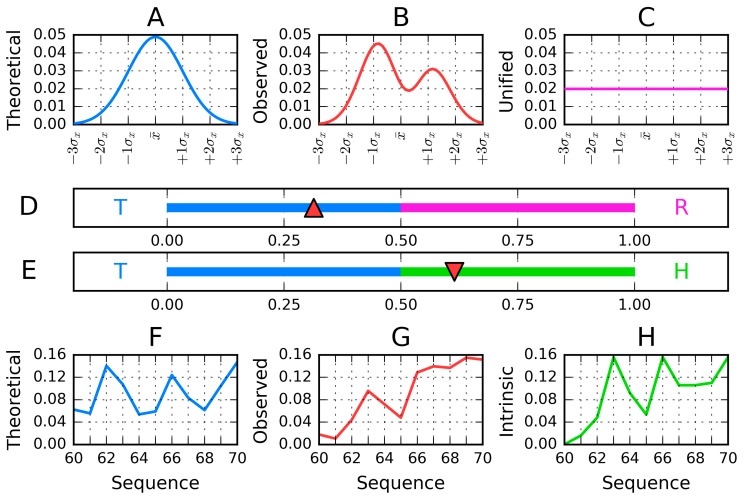
Visualization of the presented model, reduced to a single dimension for the sake of clarity: (**A**)—Gaussian distribution superimposed onto the protein molecule (T); (**B**)—Observed distribution in the molecule under consideration (O) (**C**)—Uniform distribution (R) (**D**)—Part of the Gaussian distribution plotted for the selected fragment (T_i–f_) (**E**)—Observed distribution plotted for the selected fragment (O_i–f_) (**F**)—Intrinsic distribution plotted for the selected fragment (H_i–f_) (**G**)— *RD* scale for the example illustrated in Figures (**A**–**C**,**H**)—*RD* scale for the example illustrated in Figures (**D**–**F**) (“f” indicates “fragment”).

Figure 10 provides a graphical depiction of the presented model.

The polypeptide chain folding procedure, in addition to optimizing internal free energy, also minimizes the difference between T_i_ and O_i_, directing hydrophobic residues towards the interior of the protein body, while exposing hydrophilic residues on its surface. The degree of similarity between both distributions is quantitatively expressed by a parameter referred to as Relative Distance (RD), computed in accordance with the Kullback-Leibler divergence entropy (D_KL_) formula [53]. However, in order to properly interpret the value of RD, another reference distribution is required in addition to T and O. For this reason, we introduce a distribution referred to as R, which assigns a uniform value of hydrophobicity (equal to 1/N) to each residue, where N is the number of residues in the chain. Computing *RD* for this trio of distributions (T-O-R) tells us whether the observed distribution approximates the theoretical distribution and includes a hydrophobic core (*RD* < 0.5) or is more closely aligned with the uniform distribution (*RD* ≥ 0.5). In this sense, R is treated as a “polar opposite” of T since it lacks any concentration of hydrophobicity anywhere in the protein body. In addition to the above, it is also interesting to calculate RD for a different pair of reference distributions, where R is replaced by a distribution reflecting the intrinsic hydrophobicity of each residue, denoted H. Altogether, the presented procedure yields two separate values of RD—One for the T-O-R variant and one for the T-O-H variant. These values can then be calculated either for entire chains or for selected fragments (treated as distinct structural units).

The structural properties of each input chain are also described by a set of correlation coefficients—HvT, TvO and HvO, which express pairwise differences between various distributions of hydrophobicity. In light of the presented analysis, globular proteins containing prominent hydrophobic cores should be characterized by low values of *RD* (far below 0.5) and balanced values of correlation coefficients, whereas, in amyloid proteins *RD* should remain high and the values of correlation coefficients should vary significantly (high HvO and low—Or even negative—TvO and HvT). Such conditions suggest that the given structure lacks a centralized hydrophobic core and that its conformation is determined by the individual properties (intrinsic hydrophobicity) of each participating residue. The amyloid does not “align” to the aqueous solvent and instead exhibits a linear pattern [23,24,25,26], where bands of high and low hydrophobicity alternate along the fibril’s axis. Unlike in globular proteins where the synergy between various environmental factors can be observed, amyloids are solely dependent on the intrinsic properties of their residues. This phenomenon is further discussed in Reference [22].

The models produced by Robetta, I-Tasser and FOD for the entire ASyn chain (1–140) and for its fragments (1–30, 30–100 and 100–140) have been subjected to comparative analysis based on the values of *RD* (T-O-R and T-O-H), as well as the aforementioned correlation coefficients (HvT, TvO and HvO). Similarly, to previous analyses, we identify a set of criteria which suggest susceptibility to producing amyloid structures: High values of *RD* and HvO coupled with negative values of HvT and TvO. Our comparative analysis highlights relations between individual models, as well as with regard to the structures listed in PDB (2N0A and 1XQ8).

## 5. Conclusions

Analysis of ASyn models suggests a weak preference for adopting globular conformations, with notably different properties exhibited by each fragment (1–30, 30–100 and 100–140). The FOD model generates globular structures by taking into account the active involvement of the solvent as an external force field, guiding hydrophobic residues towards the center of the protein body and exposing hydrophilic residues on its surface. This process assumes that the structure of the protein is tightly dependent on the properties of the solvent, and that—Consequently—Changes in the solvent’s properties may affect the conformations attained by polypeptide chains, as indeed experimentally observed [53]. This paper represents the part of the complex analysis of amyloid structures [23,24,25] searching for a common mechanism of amyloid formation. The model so far proposed in Reference [31] is to treat the globular structure of proteins as the result of the influence of the external force field of 3D Gauss form. However, the environment represented by 2D Gauss function promotes the structural forms observed in amyloids. The simulation of the folding process in the presence of the external force field of these two categories is currently conducted by the group. 

## Supplementary Materials

The following are available online.

## Abbreviation

ASyn	α-synuclein
