# Supplementary material for: Alternative Structures of α-Synuclein
Source: Molecules. 2020 Jan 30;25(3):600. doi: 10.3390/molecules25030600 (PMC7038196; doi:10.3390/molecules25030600)
Supplement: Supplementary file 1 [file molecules-25-00600-s001.pdf]

Supplementary Materials

Alternative Structures of α-Synuclein

Dawid Dułak [1], Małgorzata Gadzała [2], Mateusz Banach [3], Leszek Konieczny [4] and Irena Roterman [3,*]

[1] Faculty of Physics, Astronomy and Applied Computer Science, Jagiellonian University, Łojasiewicza 11, 30-348 Kraków, Poland; dawid.dulak@gmail.com
[2] ACK–Cyfronet AGH, Nawojki 11, 30-950 Krakow, Poland, currently: Schibsted Tech Polska Sp. z o.o. ul. Armii Krajowej 28, 30-150 Kraków; Poland; m.k.gadzala@gmail.com
[3] Department of Bioinformatics and Telemedicine, Jagiellonian University—Medical College, Łazarza 16, 31-530 Krakow, Poland; mateusz.banach@uj.edu.pl
[4] Chair of Medical Biochemistry, Jagiellonian University–Medical College, Kopernika 7, 31-034 Kraków, Poland; mbkoniec@cyf-kr.edu.pl
* Correspondence: correspondence: myroterm@cyf-kr.edu.pl

Table S1. Values of RD and correlation coefficients describing the micelle-bound form of ASyn (1XQ8). Top row—Entire chain (1–140); bottom row—1–95 fragment. * eliminated residues include: 36–38, 47–49, 51–53, 67–69, 85–94 (excess hydrophobicity) and 20–22 (hydrophobicity deficiency). Rows labeled "β-strands" correspond to beta folds in the amyloid structure. Rows labeled "Amyloid" represent the status of the corresponding fragments in the ASyn amyloid.

Protein Selected Fragm.	Fragment	RD		Correlation Coefficients		
		T-O-R	T-O-H	HvT	TvO	HvO
1XQ8	1–95 in 1–140	0.634	0.526	0.167	0.296	0.686
1XQ8	1–95 individual	0.646	0.539	-0.091	0.174	0.681
1XQ8	1–95* individual	0.476	0.336	0.088	0.477	0.716
1XQ8	1–140	0.644	0.601	0.095	0.261	0.687
1XQ8	37–43 In 1–140	0.817	0.401	-0.808	-0.369	0.735
β-Strands	40–56	0.370	0.271	0.220	0.699	0.671
		0.382	0.282	0.269	0.691	0.671
	60–67	0.441	0.931	0.432	0.471	0.963
		0.444	0.931	0.436	0.479	0.963
	**70–78**	**0.900**	**0.832**	**0.458**	**0.175**	**0.737**
		**0.837**	**0.739**	**0.526**	**0.542**	**0.737**
	81–84	0.529	0.392	−0.052	0.237	0.958
		**0.602**	**0.465**	**−0.400**	**−0.119**	**0.958**
	88–97	0.434	0.495	0.706	0.652	0.582
		0.495	0.361	0.018	0.709	−0.397
Helix	3–37	**0.627**	**0.410**	**−0.078**	**0.005**	**0.780**
		**0.624**	**0.407**	**−0.199**	**0.124**	**0.780**
	45–92	**0.644**	**0.681**	**0.199**	**0.302**	**0.828**
		**0.643**	**0.680**	**0.216**	**0.152**	**0.828**
Repetitive [5]	10–20	0.521	0.390	0.052	0.115	0.926
		0.447	0.323	0.318	0.468	0.929
	21–31	**0.670**	**0.587**	**−0.506**	**−0.512**	**0.913**
		**0.658**	**0.575**	**−0.416**	**−0.403**	**0.913**
	32–42	**0.732**	**0.374**	**−0.518**	**−0.267**	**0.819**
		**0.770**	**0.423**	**−0.650**	**−0.397**	**0.819**
	43–53	0.325	0.245	0.543	0.779	0.830
		0.310	0.232	0.443	0.730	0.830
	58–68	0.530	0.519	0.303	0.108	0.849
		0.530	0.519	0.350	0.155	0.849
	69–79	0.671	0.816	0.524	0.251	0.768
		0.510	0.694	0.638	0.587	0.768
	80–90	0.365	0.271	0.338	0.695	0.765
		**0.794**	**0.713**	**−0.692**	**−0.838**	**0.765**
	10–90	**0.605**	**0.533**	**0.121**	**0.321**	**0.812**
		**0.605**	**0.533**	**−0.002**	**0.137**	**0.812**
Discussed Parkinson [5]	**25–35**	**0.554**	**0.370**	**−0.052**	**0.120**	**0.939**
		**0.545**	**0.362**	**0.021**	**0.195**	**0.939**
[5]	26–98	**0.675**	**0.644**	**0.131**	**0.210**	**0.746**
		**0.655**	**0.589**	**−0.020**	**0.201**	**0.706**

[5]	1–25	**0.501**	**0.289**	**−0.066**	**0.225**	**0.633**
		**0.568**	**0.347**	**−0.180**	**0.296**	**0.633**
Amyloid	38–42	**0.688**	**0.640**	**−0.616**	**0.303**	**0.357**
		**0.625**	**0.574**	**−0.461**	**0.445**	**0.357**
	68–82	**0.758**	**0.691**	**−0.132**	**−0.418**	**0.739**
		**0.612**	**0.529**	**0.044**	**0.123**	**0.739**
	78–85	0.501	0.335	0.100	0.388	0.806
		**0.647**	**0.478**	**−0.529**	**−0.424**	**0.806**
	67–82	**0.768**	**0.703**	**−0.123**	**−0.443**	**0.721**
		**0.617**	**0.535**	**0.046**	**0.099**	**0.721**
	80–85	0.440	0.240	0.046	0.505	0.785

Table S2. RD values and correlation coefficients for the ASyn amyloid. A—Only cross-chain interactions B—30–100 fragment treated as part of the complex; C—30–100 fragment treated as a standalone unit; D—30–100 fragment treated as a standalone unit with residues 43–46, 50, 79, 80 and 83 eliminated; E—Fragments treated as part of the complex; F—Fragments treated as part of the complex with residues 80–83 eliminated.

N0A	FRAGMENT	RD		Correlation Coefficients		
		T-O-R	T-O-H	HvT	TvO	HvO
	1–140	0.472	0.498	0.141	0.576	0.666
	1–140	0.485	0.687	0.172	0.303	0.884
	1–30	0.665	0.479	−0.043	0.222	0.611
	30–100	0.477	0.547	0.169	0.339	0.821
	30–100	0.531	0.598	0.242	0.493	0.798
	30–100	0.493	0.581	0.359	0.632	0.766
	100–140	0.683	0.601	0.000	0.189	0.709
	1–100	0,512	0,513	0,106	0,506	0,703
	1–100	0,494	0,497	0,143	0,565	0,694
	30–100	0,455	0,529	0,233	0,434	0,816
	(1–30)+ (100–140)	0,686	0.569	−0.050	0.091	0.644

Table S3. Values of RD and correlation coefficients describing the amyloid form of ASyn (2N0A). Top row—Entire chains (1–140); bottom row—30–100 fragments. * eliminated residues include: 37, 43–46 and 80. Rows labeled "Beta" correspond to corresponds to chain A. Rows labeled "Amyloid" represent the status of the corresponding fragments in the ASyn amyloid.

Protein Selected Fragm.	Fragment	RD		Correlation Coefficients		
		T-O-R	T-O-H	HvT	TvO	HvO
2N0A	1–140	0.472	0.498	0.141	0.576	0.666
2N0A	30–100	0.531	0.598	0.241	0.492	0.798
2N0A*	30–100*	0.496	0.581	0.359	0.632	0.766
2N0A	37–43	0.528	0.382	−0.298	−0.107	0.808
	In 1–140	0.706	0.570	−0.596	−0.393	0.808
Beta	**38–55**	**0.599**	**0.551**	**−0.386**	**−0.395**	**0.839**
		**0.597**	**0.548**	**−0.400**	**−0.191**	**0.839**
	61–66	0.387	0.802	0.469	0.781	0.894
		0.436	0.832	0.257	0.583	0.894
	**70–78**	**0.410**	**0.473**	**0.056**	**0.626**	**0.723**
		**0.607**	**0.690**	**−0.205**	**0.256**	**0.723**
	81–83	0.487	0.604	0.958	0.929	0.996

		**0.331**	**0.443**	**0.791**	**0.733**	**0.996**
	88–97	0.460	0.541	0.498	0.658	0.799
		0.266	0.336	0.404	0.834	0.799
Helix	45–92	0.466	0.630	0.176	0.380	0.843
		0.496	0.658	0.253	0.439	0.843
Outside of Complex	45–92	0.472	0.653	0.177	0.357	0.874
		0.468	0.648	0.322	0.432	0.874
Repetitiv	32–42	0.419	0.254	0.519	0.617	0.848
		0.434	0.286	0.502	0.611	0.853
	43–53	0.540	0.580	−0.213	−0.181	0.916
		0.482	0.523	0.208	0.347	0.916
	58–68	0.349	0.502	0.578	0.886	0.830
		0.392	0.547	0.384	0.384	0.830
	**69–79**	**0.551**	**0.697**	**−0.297**	**0.050**	**0.730**
		**0.643**	**0.771**	**−0.083**	**0.208**	**0.730**
	**80–90**	**0.508**	**0.578**	**−0.078**	**−0.028**	**0.784**
		**0.580**	**0.647**	**0.057**	**0.111**	**0.784**
	32–90	0.464	0.517	0.198	0.375	0.835
		0.522	0.583	0.259	0.447	0.836
Discussed Parkinson [5]	25–35	0.635	0.358	−0.548	−0.414	0.794
		**0.759**	**0.374**	**−0.504**	**−0.463**	**0.874**
[5]	30–98	0.477	0.551	0.175	0.339	0.840
		0.545	0.622	0.256	0.471	0.835
Amyloid [5]	38–42	**0.586**	**0.348**	**−0.307**	**0.023**	**0.117**
		**0.846**	**0.672**	**−0.484**	**0.096**	**0.117**
	68–82	**0.581**	**0.666**	**−0.320**	**−0.338**	**0.748**
		**0.587**	**0.671**	**−0.041**	**0.119**	**0.748**
	78–85	**0.510**	**0.577**	**−0.080**	**−0.084**	**0.826**
		**0.586**	**0.650**	**−0.071**	**0.036**	**0.826**
	67–82	**0.586**	**0.537**	**−0.299**	**−0.343**	**0.740**
		**0.609**	**0.689**	**−0.032**	**0.090**	**0.740**
	80–85	**0.509**	**0.537**	**−0.051**	**−0.082**	**0.853**

Table S4. RD values and correlation coefficients for models generated by the presented software packages for the 1–140 sequence of ASyn. Values listed in boldface correspond to RD ≥ 0.5.

**1–140 Fragment**							
**RD**	**Correlation Coefficient**				**Model**		
**TOR**	**TOH**	**HvT**	**TvO**	**HvO**	**Model**	**Secondary**	**Globular**
**Micelle-bound Structure Listed In PDB (1XQ8)**							
**0.644**	**0.601**<br>**0.095**		**0.261**	**0.687**	**1XQ8—PDB**	**Helix + RD**	**F**
					**Fuzzy Oil Drop**		

**0.525**	0.392	**0.107**	**0.394**	**0.579**	**FOD_1_(202)**	**Helix +RC**	**T**
**0.558**	**0.400**	**0.093**	**0.425**	**0.599**	**FOD_2_(081)**	**Helix + RC**	**T**
**0.567**	**0.468**	**0.016**	**0.208**	**0.594**	**FOD_3_(376)**	**RC**	**T**
**0.575**	**0.416**	**−0.017**	**0.277**	**0.613**	**FOD_4_(038)**	**Helix + RC**	**T**
**0.583**	**0.438**	**0.108**	**0.315**	**0.617**	**FOD_5_(328)**	**RC + Helix**	**T**
						**I-Tasser**	
**0.592**	0.437	**0.186**	**0.462**	**0.632**	**ITASSER_1_(1)**	**Helix + RC (BZ)**	**F**
						**Robetta**	
**0.574**	**0.567**	**0.088**	**0.308**	**0.693**	**ROBETTA_1_(2)**	**Helix + RC**	**F**
**0.585**	**0.538**	**0.135**	**0.401**	**0.705**	**ROBETTA_2_(3)**	**Helix + RC BZ**	**F**
**0.651**	**0.658**	**0.153**	**0.348**	**0.779**	**ROBETTA_3_(1)**	**Helix**	**F**
**0.676**	**0.622**	**0.160**	**0.425**	**0.699**	**ROBETTA_4_(5)**	**Helix + RC BZ**	**F**

Table S5. RD values and correlation coefficients for models generated by the presented software packages for the 1–30 sequence of ASyn. Values listed in boldface correspond to RD ≥ 0.5.

				**1–30 Fragment**			
**RD**	**Correlation Coefficient**				**Model**		
**TOR**	**TOH**	**HvT**	**TvO**	**HvO**	**Model**	**Secondary**	**Globular**
				**Fuzzy Oil Drop**			
0.195	0.056	0.025	0.869	0.329	FOD_1_(053)	Helix	T
0.198	0.075	0.113	0.867	0.423	FOD_2_(170)	Helix	T
0.198	0.081	0.005	0.857	0.318	FOD_3_(295)	Helix	T
0.202	0.104	0.364	0.893	0.529	FOD_4_(281)	Helix	T
0.202	0.073	0.155	0.889	0.364	FOD_5_(328)	Helix	T
				**I-Tasser**			
0.399	0.207	0.246	0.661	0.653	ITASSER_1_(3)	Helix	T
**0.595**	**0.295**	**0.082**	**0.338**	**0.627**	**ITASSER_2_(2)**	**Helix**	**F**

**0.701**	**0.378**	**-0.086**	**0.299**	**0.611**	**ITASSER_3_(1)**	**Helix**	**F**
					**Robetta**		
**0.623**	**0.433**	**0.012**	**0.388**	**0.771**	**ROBETTA_1_(4)**	**Helix**	**F**
**0.623**	**0.428**	**0.014**	**0.395**	**0.775**	**ROBETTA_2_(2)**	**Helix**	**F**
**0.624**	**0.433**	**0.020**	**0.395**	**0.769**	**ROBETTA_3_(3)**	**Helix**	**F**
**0.628**	**0.437**	**0.018**	**0.392**	**0.772**	**ROBETTA_4_(5)**	**Helix**	**F**

Table S6. Summary of parameters reflecting the presence/absence of centralized hydrophobic cores in models produced by FOD, I-Tasser and Robetta. Values listed in boldface diverge from the theorized globular structure. T and F indicate "true" and "false" respectively. The presented analysis concerns the 1–30 fragment. RD values and correlation coefficients for models generated by the presented software packages for the 1–30 sequence of ASyn. Values listed in boldface correspond to RD ≥ 0.5.

					**30–100 Fragment**		
**RD**		**Correlation Coefficient**				**Model**	
**TOR**	**TOH**	**HvT**	**TvO**	**HvO**	**Model**	**Secondary**	**Globular**
					**Fuzzy Oil drop**		
0.483	0.361	0.244	0.496	0.660	FOD_1_(289)	RC + Helix	T
**0.511**	**0.399**	**0.004**	**0.412**	**0.606**	**FOD_2_(058)**	**Helix + RC**	**T**
**0.514**	**0.403**	**0.105**	**0.404**	**0.592**	**FOD_3_(111)**	**Helix + RC**	**T**
**0.520**	**0.411**	**-0.071**	**0.354**	**0.544**	**FOD_4_(199)**	**Helix + RC**	**T**
**0.521**	**0.412**	**0.065**	**0.388**	**0.593**	**FOD_5_(347)**	**Helix + RC**	**T**
					**I-Tasser**		
**0.631**	**0.519**	**0.204**	**0.355**	**0.596**	**Itasser_1_(1)**	**Helix**	**F**
					**Robetta**		
0.295	0.336	0.543	0.772	0.777	ROBETTA_1_(2)	Helix + Beta	T
**0.514**	**0.530**	**0.192**	**0.470**	**0.754**	**ROBETTA_2_(1)**	**Helix + Beta**	**T**
**0.514**	**0.434**	**0.056**	**0.483**	**0.691**	**ROBETTA_3_(3)**	**Helix**	**T**

**0.655**	**0.591**	**0.221**	**0.237**	**0.713**	**ROBETTA_4_(4)**	**Helix**	**F**
**0.662**	**0.567**	**0.175**	**0.248**	**0.707**	**ROBETTA_5_(5)**	**Helix**	**F**

Table S7. Quantitative characterization of models produced by FOD, I-Tasser and Robetta for the 100–140 fragment. The presented values determine to what extent each model approximates a spherical micelle with a centralized hydrophobic core. Values listed in boldface indicate discordance vs. the theoretical model. "T" and "F" indicate true and false respectively. RD values and correlation coefficients are listed for models generated by the presented software packages for the 1–30 fragment of ASyn. Values listed in boldface correspond to RD ≥ 0.5.

**100–140 Fragment**							
**RD**	**Correlation Coefficient**				**Model**		
**TOR**	**TOH**	**HvT**	**TvO**	**HvO**	**Model**	**Secondary**	**Globular**
					**Fuzzy Oil Drop**		
0.299	0.208	0.205	0.746	0.440	FOD_1_(002)	RC + Helix	T
0.326	0.215	0.157	0.728	0.399	FOD_2_(170)	RC + Helix	T
0.331	0.247	0.155	0.706	0.533	FOD_3_(088)	RC + Helix	T
0.332	0.310	0.227	0.672	0.561	FOD_4_(034)	RC + Helix	T
0.333	0.266	0.242	0.729	0.519	FOD_5_(020)	Helix + RC	T
					**I-Tasser**		
0.317	0.393	0.510	0.727	0.816	ITASSER_1_(5)	RC	T
0.326	0.384	0.506	0.713	0.803	ITASSER_2_(3)	RC + Helix	T
0.366	0.457	0.436	0.648	0.828	ITASSER_3_(1)	RC + Helix	T
0.378	0.376	0.385	0.610	0.687	ITASSER_4_(4)	RC + Helix	T
0.491	0.297	0.154	0.571	0.539	ITASSER_5_(2)	RC	T
					**Robetta**		
0.325	0.453	0.586	0.743	0.866	ROBETTA_1_(1)	Helix (3)	F
0.362	0.430	0.371	0.629	0.775	ROBETTA_2_(4)	B + RC +Helix	F
0.383	0.442	0.345	0.623	0.810	ROBETTA_3_(5)	Beta + Helix	F
**0.556**	**0.496**	**0.271**	**0.271**	**0.689**	**ROBETTA_4_(2)**	**RC + Helix**	**F**

**0.614**	**0.537**	**0.200**	**0.200**	**0.593**	**ROBETTA_5_(3)**	**RC + Helix**	**F**

Table S8. Status of ASyn chain fragments identified as amyloidogenic (listed in bold).

Protein Selected Fragm.	FRAGMENT	RD		CORRELATION COEFFICIENTS		
		T-O-R	T-O-H	HvT	TvO	HvO
Parkinson's [5]						
FOD_1	30–35	0.407	0.055	−0.087	0.562	0.577
FOD_2		**0.687**	0.103	**−0.351**	**0.114**	**0.634**
FOD_3		**0.566**	0.056	**−0.375**	**−0.778**	**0.560**
FOD_4		**0.584**	0.214	**−0.009**	**0.230**	**0.810**
FOD_5		**0.594**	0.200	**−0.177**	**−0.046**	**0.744**
I-TASSER		**0.766**	0.204	**−0.547**	**−0.717**	**0.870**
Robetta_1		**0.732**	0.263	**−0.557**	**0.278**	**0.494**
Robetta_2		0.253	0.061	0.684	0.752	0.742
Robetta_3		0.211	0.103	0.664	0.898	0.807
Robetta_4		**0.543**	0.148	−0.370	0.233	0.558
Robetta_5		**0.635**	0.094	−0.174	0.092	0.706
Fragment discussed in [5]						
FOD_1	30–98	0.495	0.367	0.241	0.481	0.692
FOD_2		**0.532**	**0.413**	**0.016**	**0.380**	**0.642**
FOD_3		**0.521**	**0.403**	**0.088**	**0.407**	**0.629**
FOD_4		**0.539**	**0.423**	**-0.080**	**0.324**	**0.576**
FOD_5		**0.531**	**0.420**	**0.082**	**0.376**	**0.624**
I-TASSER		**0.644**	**0.530**	**0.217**	**0.317**	**0.628**
Robetta_1		0.299	0.338	0.531	0.767	0.781
Robetta_2		**0.527**	**0.548**	**0.205**	**0.447**	**0.787**
Robetta_3		**0.540**	**0.454**	**0.063**	**0.448**	**0.733**
Robetta_4		**0.672**	**0.610**	**0.235**	**0.196**	**0.759**
Robetta_5		**0.677**	**0.583**	**0.189**	**0.205**	**0.753**
Amyloid [5]	38–42					
FOD_1		**0.554**	**0.507**	0.408	0.548	0.423
FOD_2		0.450	0.289	−0.588	0.701	−0.296
FOD_3		0.471	0.310	0.180	0.327	−0.279
FOD_4		0.469	0.297	−0.525	0.423	−0.675
FOD_5		0.331	0.221	0.324	0.650	−0.349
I-TASSER		0.431	0.318	−0.638	0.573	−0.081
Robetta_1		**0.647**	**0.613**	0.379	0.667	0.393
Robetta_2		0.092	0.261	0.883	0.995	0.862
Robetta_3		0.386	0.388	−0.075	0.603	0.330
Robetta_4		0.401	0.516	0.390	0.551	0.632
Robetta_5		**0.529**	0.252	−0.536	0.636	−0.483
**[5]**	67–82					
FOD_1		**0.669**	**0.646**	**0.280**	**0.288**	**0.624**
FOD_2		**0.612**	**0.520**	**0.039**	**0.425**	**0.596**
FOD_3		**0.632**	**0.588**	**0.064**	**0.335**	**0.580**
FOD_4		**0.747**	**0.536**	**0.116**	**0.056**	**0.672**

FOD_5		**0.661**	**0.543**	**0.041**	**0.318**	**0.584**
I-TASSER		0.495	0.333	0.302	0.271	0.487
Robetta_1		0.344	0.426	0.535	0.690	0.841
Robetta_2		**0.716**	**0.671**	**−0.277**	**−0.093**	**0.625**
Robetta_3		**0.698**	**0.747**	**0.000**	**0.302**	**0.762**
Robetta_4		0.293	0.210	0.441	0.821	0.589
Robetta_5		0.313	0.226	0.334	0.803	0.588
**[5]**	78–85					
FOD_1		0.205	0.183	0.664	0.885	0.698
FOD_2		0.471	0.377	0.499	0.312	0.848
FOD_3		0.401	0.279	0.449	0.493	0.781
FOD_4		0.420	0.204	0.707	0.456	0.750
FOD_5		0.423	0.295	0.696	0.508	0.872
I-TASSER		**0.618**	**0.417**	**−0.381**	**−0.600**	**0.620**
Robetta_1		0.280	0.381	0.837	0.832	0.843
Robetta_2		0.468	0.304	0.058	0.507	0.639
Robetta_3		0.348	0.441	0.696	0.706	0.915
Robetta_4		**0.551**	**0.471**	**0.080**	**−0.044**	**0.767**
Robetta_5		**0.579**	**0.496**	**−0.121**	**−0.284**	**0.767**
**[5]**	68–82					
FOD_1		**0.676**	**0.653**	**0.312**	**0.216**	**0.650**
FOD_2		**0.646**	**0.548**	**0.055**	**0.289**	**0.671**
FOD_3		**0.669**	**0.625**	**0.070**	**0.270**	**0.666**
FOD_4		**0.748**	**0.525**	**0.114**	**0.094**	**0.715**
FOD_5		**0.663**	**0.544**	**0.041**	**0.319**	**0.591**
I-TASSER		0.491	0.320	0.302	0.302	0.529
Robetta_1		0.347	0.429	0.539	0.685	0.845
Robetta_2		**0.723**	**0.678**	**−0.279**	**−0.110**	**0.646**
Robetta_3		**0.700**	**0.750**	**0.002**	**0.298**	**0.769**
Robetta_4		0.315	0.223	0.502	0.788	0.645
Robetta_5		0.335	0.239	0.392	0.765	0.645
**[5]**	80–85					
